# Dynamic Amodal Completion Through the Magic Wand Illusion

**DOI:** 10.1177/2041669519895028

**Published:** 2019-12-27

**Authors:** Christopher W. Tyler

**Affiliations:** Division of Optometry, City University of London, UK

**Keywords:** illusions, amodal completion, perceptual organization, contours/surfaces, motion

## Abstract

In the Magic Wand effect, an overlying figure of the same color as its background is revealed by the motion of a wand *behind* it. The occluding figure is inferred by integration of the occluding edge information over time. The overlying figure is perceived by modal completion, while the wand and the background underneath are perceived by amodal completion. This illusion is compared with its predecessor from nearly two centuries ago, the Plateau Anorthoscopic Illusion, in which an object is recognizable when moved behind a slit.

This article provides an analysis of the Magic Wand illusion (Figure 1), in which an object is revealed relative to its background by a *Magic Wand* waving behind the object region but in front of its background region (see Tyler, 2011). At any given moment, only a small part of the object is revealed in this way, but the motion of the wand carries it around all parts of the object, allowing the whole structure to be completed by cumulation over time. In the terms developed by Michotte, Thinès, and Crabbé (1964), the overlying triangle is perceived by *modal completion* (or illusory perception of the overlying implied object), while the hidden part of the wand and the background underneath it are perceived by *amodal completion* (or perception of the spatial configuration of the implied object without perception of its modal properties such as color; Scherzer & Ekroll, 2015).

**Figure 1.**
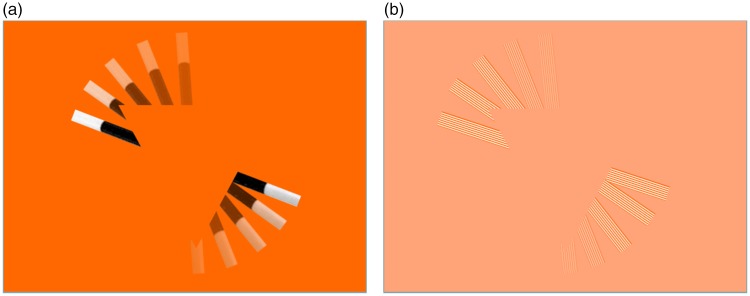
The Magic Wand revealing an equichromatic triangle occluding it. (a and Film Clip I) The triangle structure is revealed by its local occlusion of the Magic Wand bar as it moves behind the figure (with the movement depicted by the fading wand). (b and Film Clip II) The same configuration with a striped bar equiluminant with the background, to avoid leaving a retinal afterimage as it moves. The foreground/background color thus has to have half the contrast of the original (see Supplemental material).

In this form, the revealed shape could be carried by retinal persistence of the edge information. If the eyes maintain fixation at any point in the field, the edge contours will build up over time on the retina. With sufficient persistence, the entire outline could build up as a brightening luminance retinal afterimage. (Note that the actual appearance is of a *dark shadow* induced on the inside of the triangle near the wand as it moves, with only a minimum of the predicted *afterimage brightening* in the region just vacated by the wand.)

To determine whether these luminance-induced effects are a significant factor in the illusion, a version with equiluminant stripes in the wand is depicted in Figure 1(b). Now the retinal afterimage in each stripe of the moving bar is canceled by the following stripe, leaving no net afterimage. Only some form of cortical persistence of the second-order contrast modulation could provide the information for building up the occluding structure. Observation of this condition in Film Clip II makes it clear that the perception of the triangle is just as strong as with the first-order luminance wand, and thus that that it reveals a true modal/amodal completion mechanism without the aid of a retinal afterimage (see Supplemental material).

A further elaboration of the effect was a finalist in the 2011 Best Illusion of the Year contest (Tyler, 2011). This version used a triplet of three nonintersecting lines as the seed for completion of an Illusory Impossible Triangle figure (Penrose & Penrose, 1958, Film Clip III). In themselves, the three lines specify only a flat, unambiguous triangular figure (Figure 2(a)). However, in combination with the solid block triangle figure elicited by the moving wand, the depth-ambiguous Impossible Triangle is revealed (Figure 2(b), Film Clip IV). Any one vertex of the triangle has a defined depth structure, but each is incompatible with the depth structure of the other two, so the depth rotates according to which vertex is being fixed at any given time. The same impression of an illusory Impossible Triangle is elicited by the occlusion of three spheres in the Supplemental Material (Film Clip V), designed to evoke the concept of the modal/amodal completion principles of the Kanizsa Triangle in combination with the Impossible Triangle. These two versions therefore show the Magic Wand effect giving rise to the dynamic Illusory Impossible Triangle.

**Figure 2.**
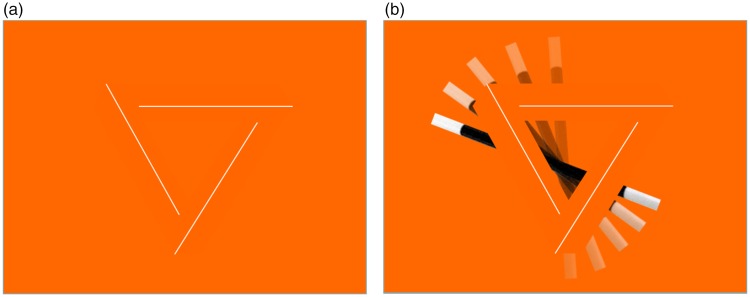
(a and Film Clip IV) The inner edges of the Penrose Impossible Triangle demarcated by white lines that by themselves carry no 3D structure information. (b and Film Clip V) The Magic Wand revealing the impossible illusory triangle in which the white lines are embedded. It is only in the context of the dynamic orange outline that the Penrose impossible triangle structure is revealed.

## Relation to the Plateau illusion

As early as 1829, Jean Plateau described a dynamic form of amodal completion that was a literal form of the Biblical metaphor of the “camel passing through the eye of a needle.” A silhouette (*the camel*) is passed behind a narrow vertical slit (*the needle*), such that the viewer only sees the upper and lower boundary points through the slit at any given moment in time. Cumulation of their positions over time can recover the full profile of the silhouette in perception, even though it never existed on the retina, constituting a dynamic form of amodal completion developed before the concept of amodal completion had been enunciated by Michotte et al. (1964) over a century later. Plateau’s focus was on the compressive distortion of the form perceived under these conditions (termed the *anorthoscopic effect*), but no such distortion is evident in the inverse version described here, underlining a core difference between the two effects.

## Supplemental Material

IPE895028 Supplemental Material - Supplemental material for Dynamic Amodal Completion Through the Magic Wand Illusion Click here for additional data file. Supplemental material, IPE895028 Supplemental Material for Dynamic Amodal Completion Through the Magic Wand Illusion by Christopher W. Tyler in i-Perception

## Supplemental Material

IPE895028 MagicWandMovie3 - Supplemental material for Dynamic Amodal Completion Through the Magic Wand Illusion Click here for additional data file. Supplemental material, IPE895028 MagicWandMovie3 for Dynamic Amodal Completion Through the Magic Wand Illusion by Christopher W. Tyler in i-Perception
