# Supplementary figures and images for: Dynamic Amodal Completion Through the Magic Wand Illusion
Source: Iperception. 2019 Dec 27;10(6):2041669519895028. doi: 10.1177/2041669519895028 (PMC6937430; doi:10.1177/2041669519895028)

## Slide 1
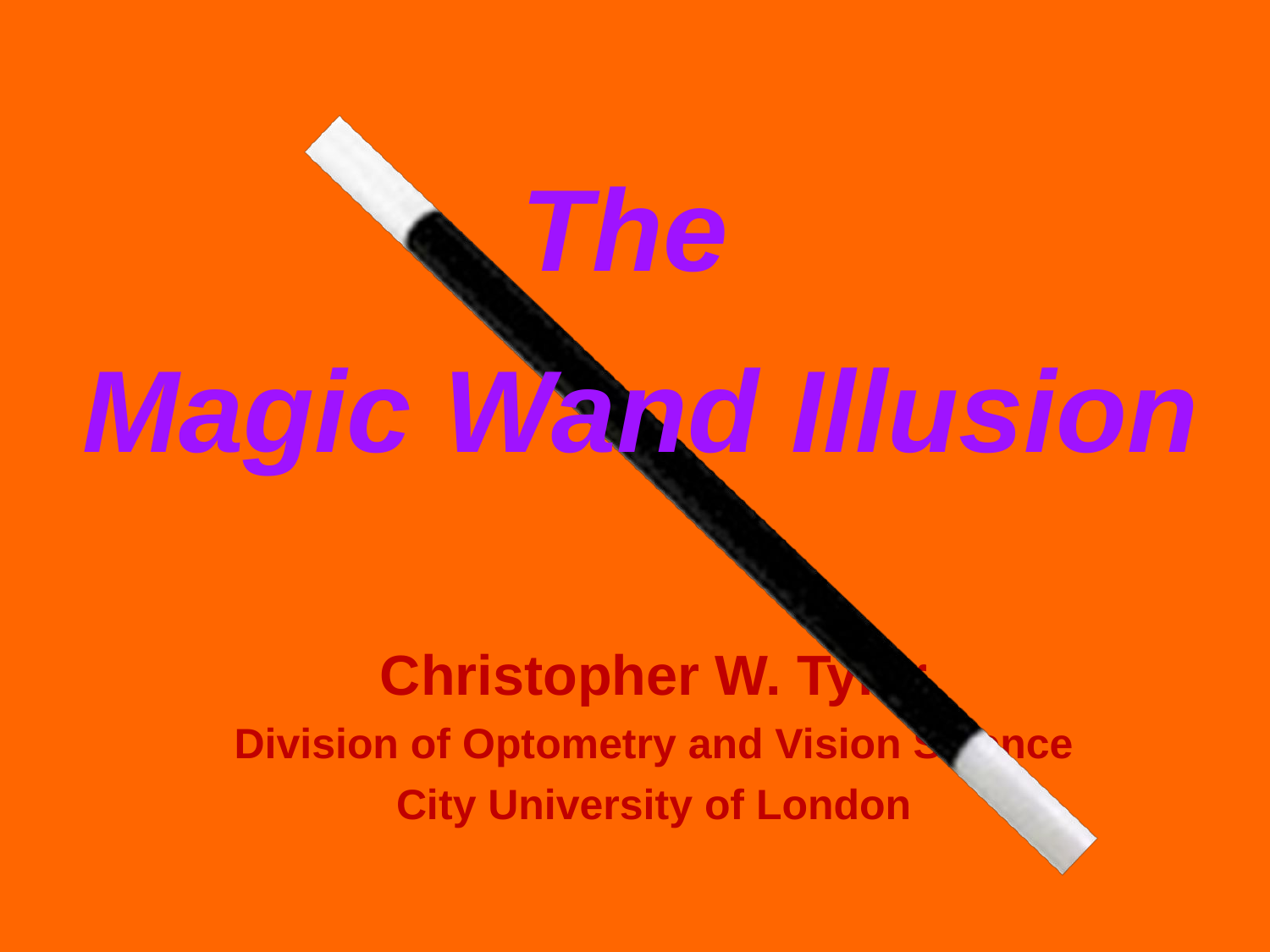

## Slide 2
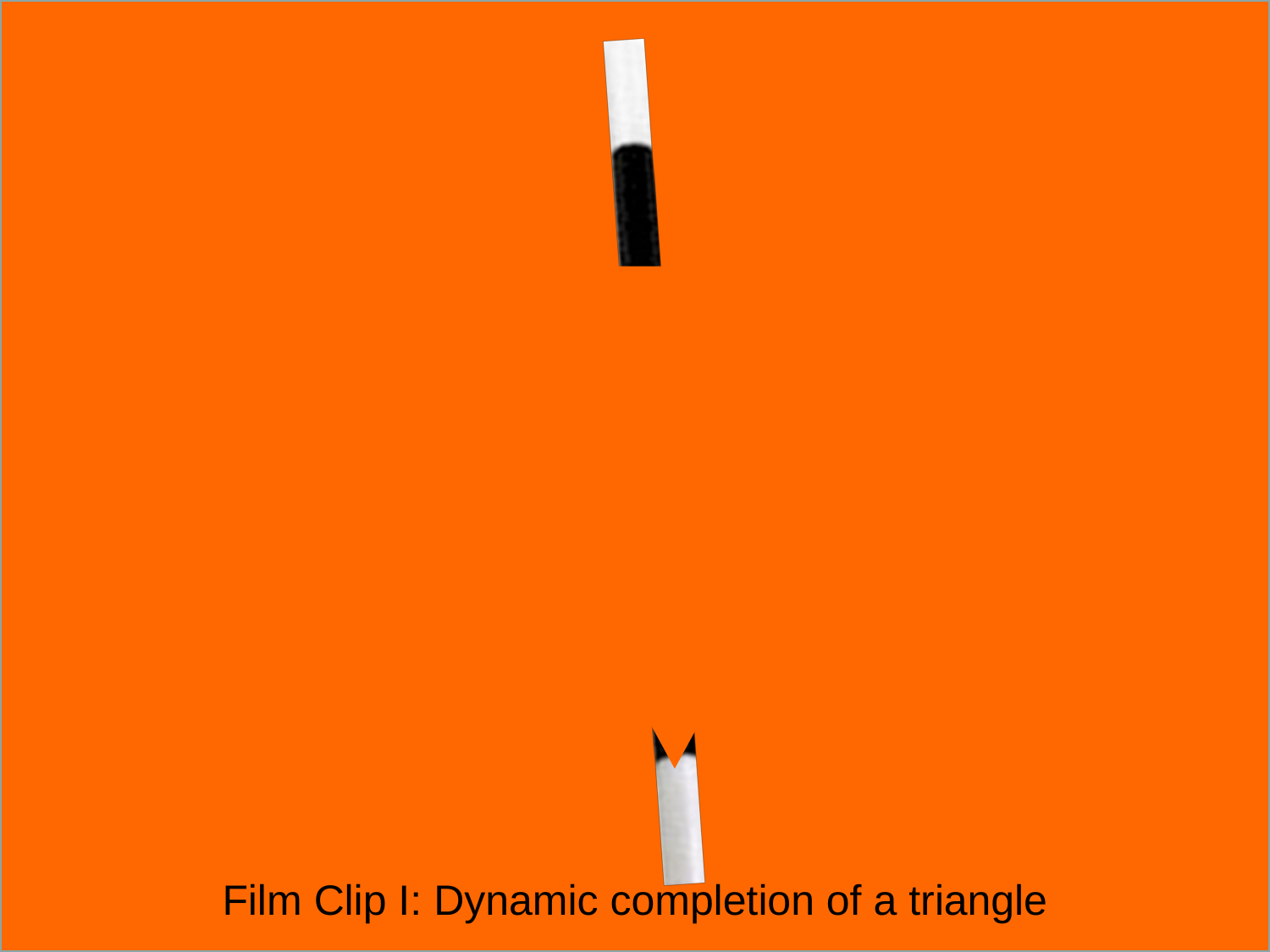

## Slide 3
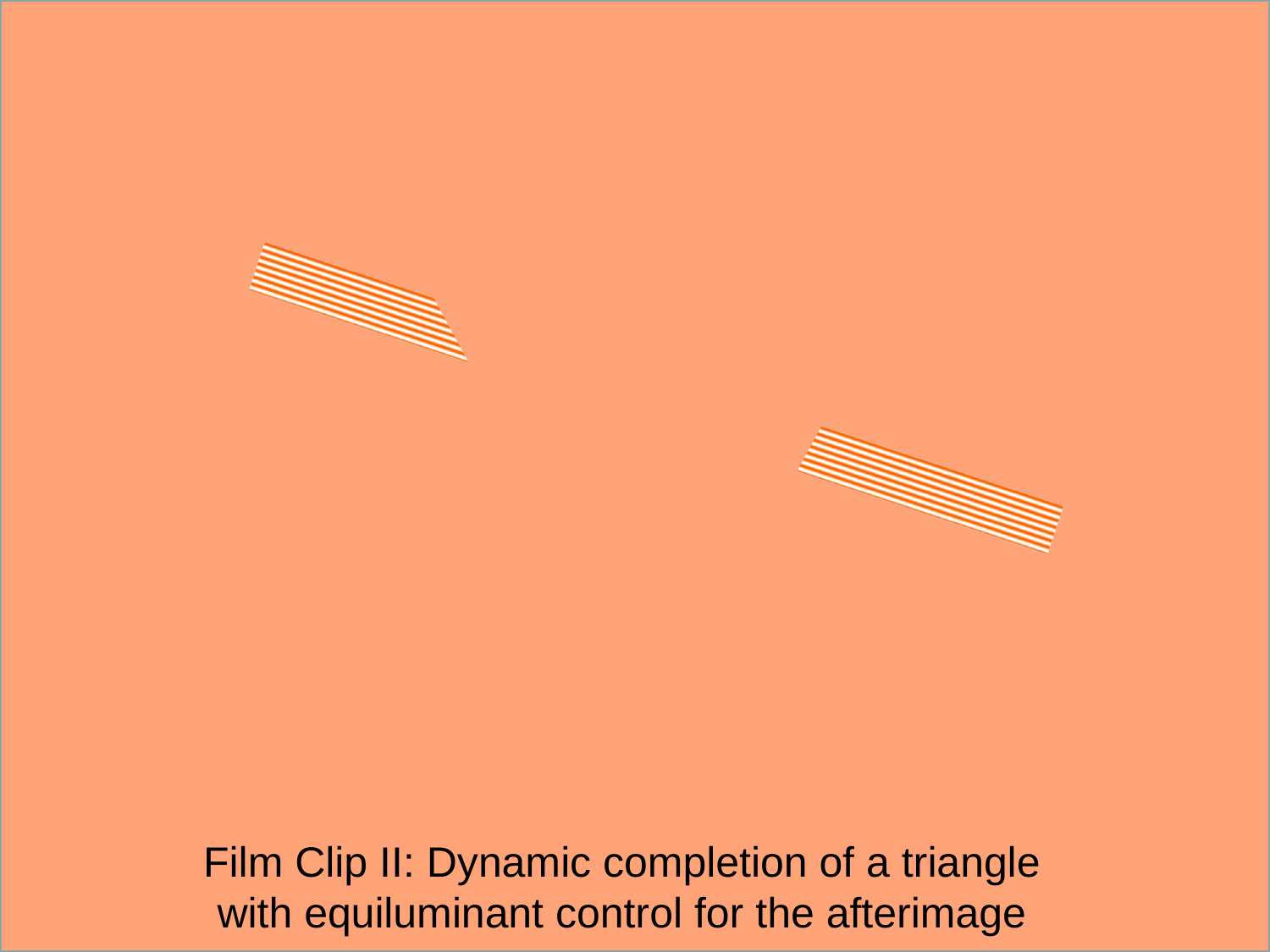

## Slide 4
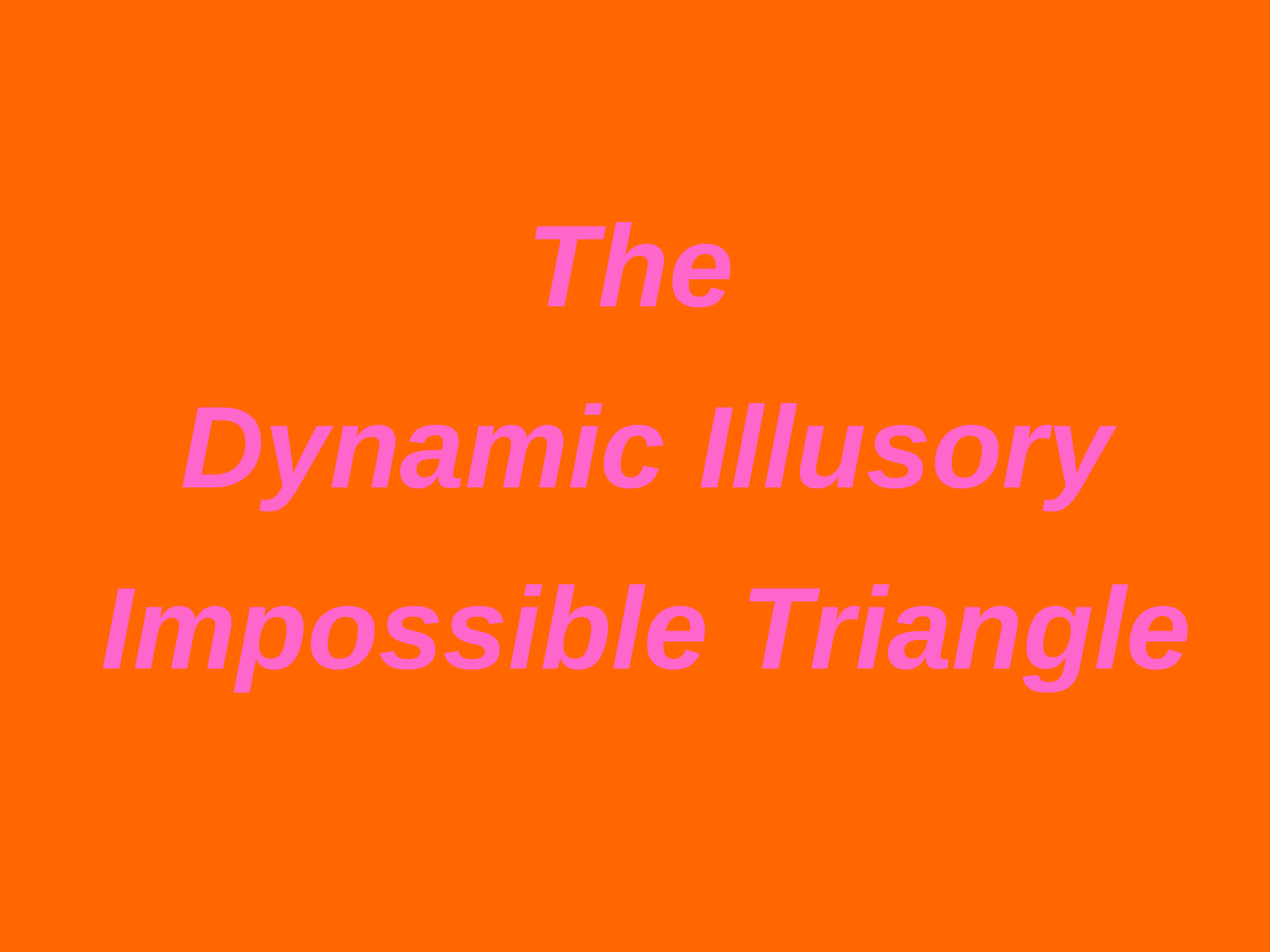

## Slide 5
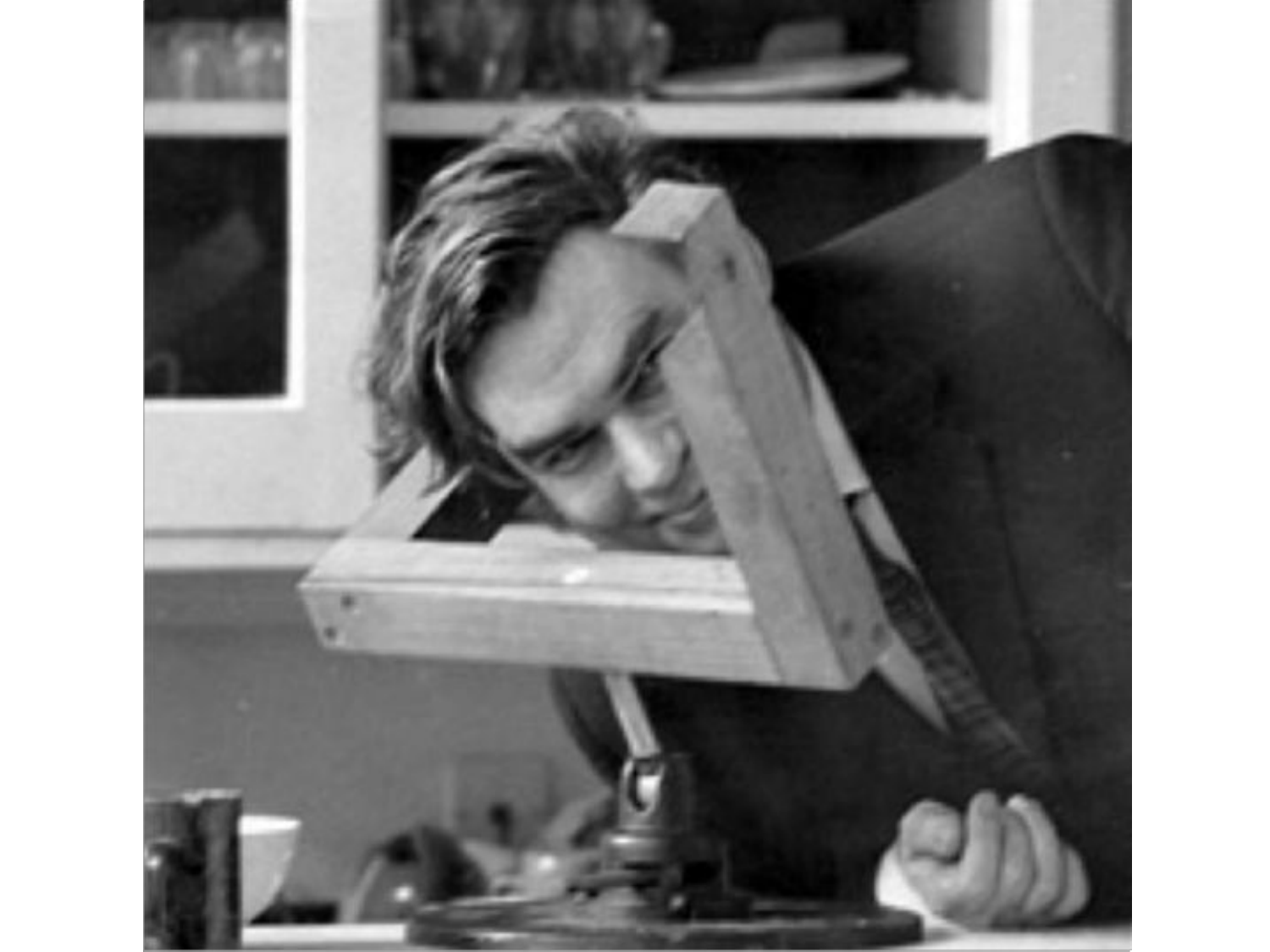

## Slide 6
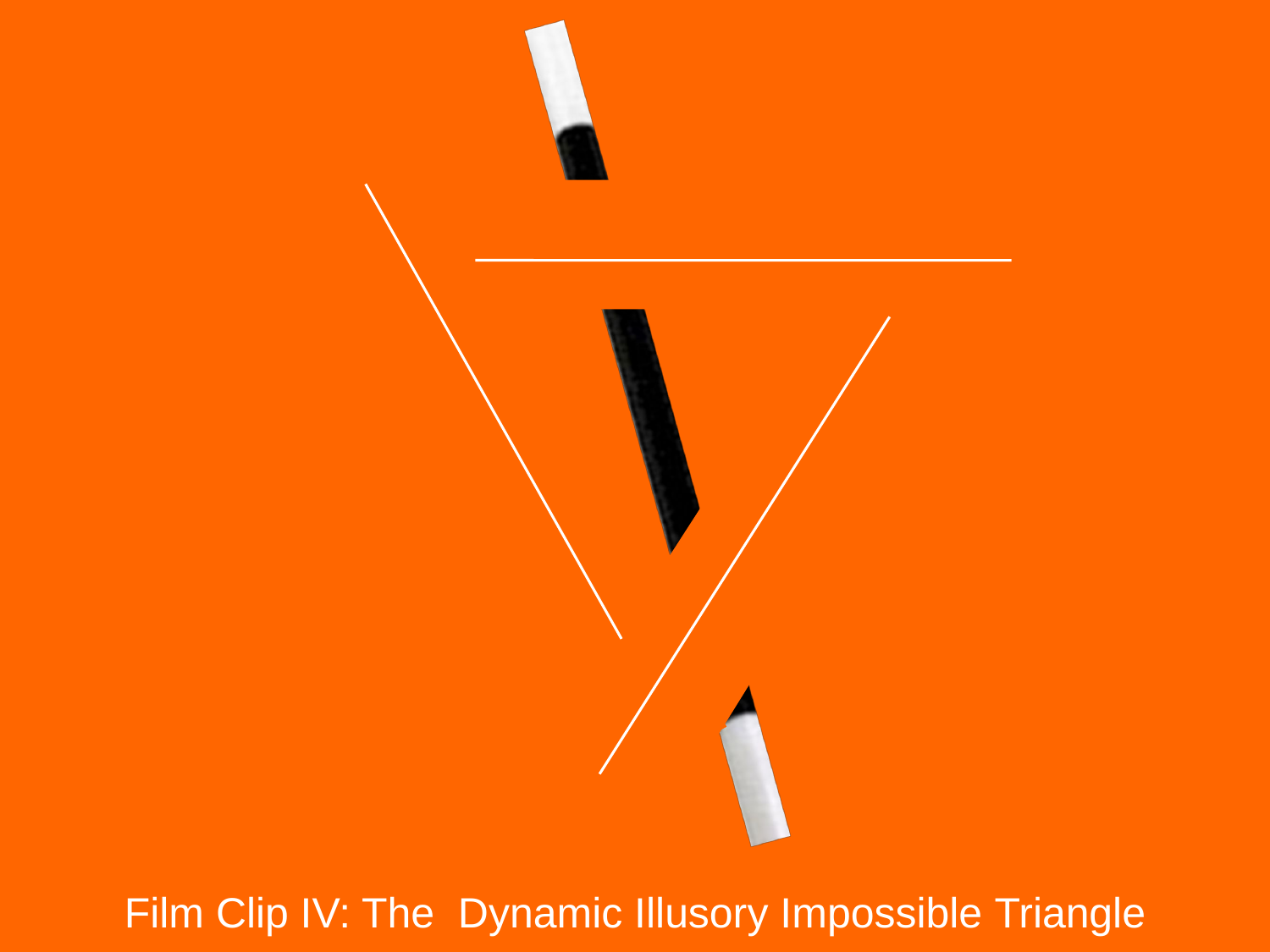

## Slide 7
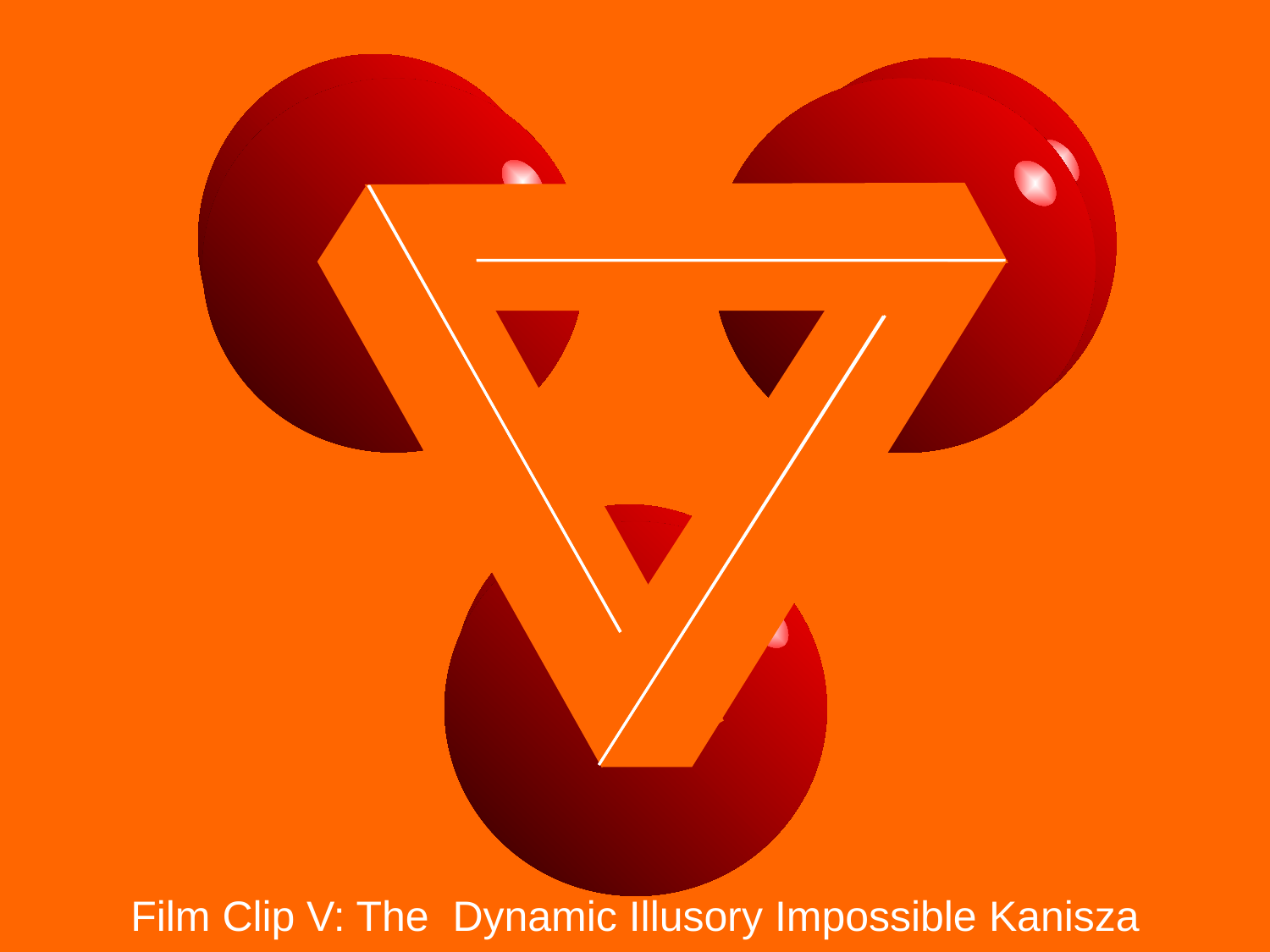

## Slide 8
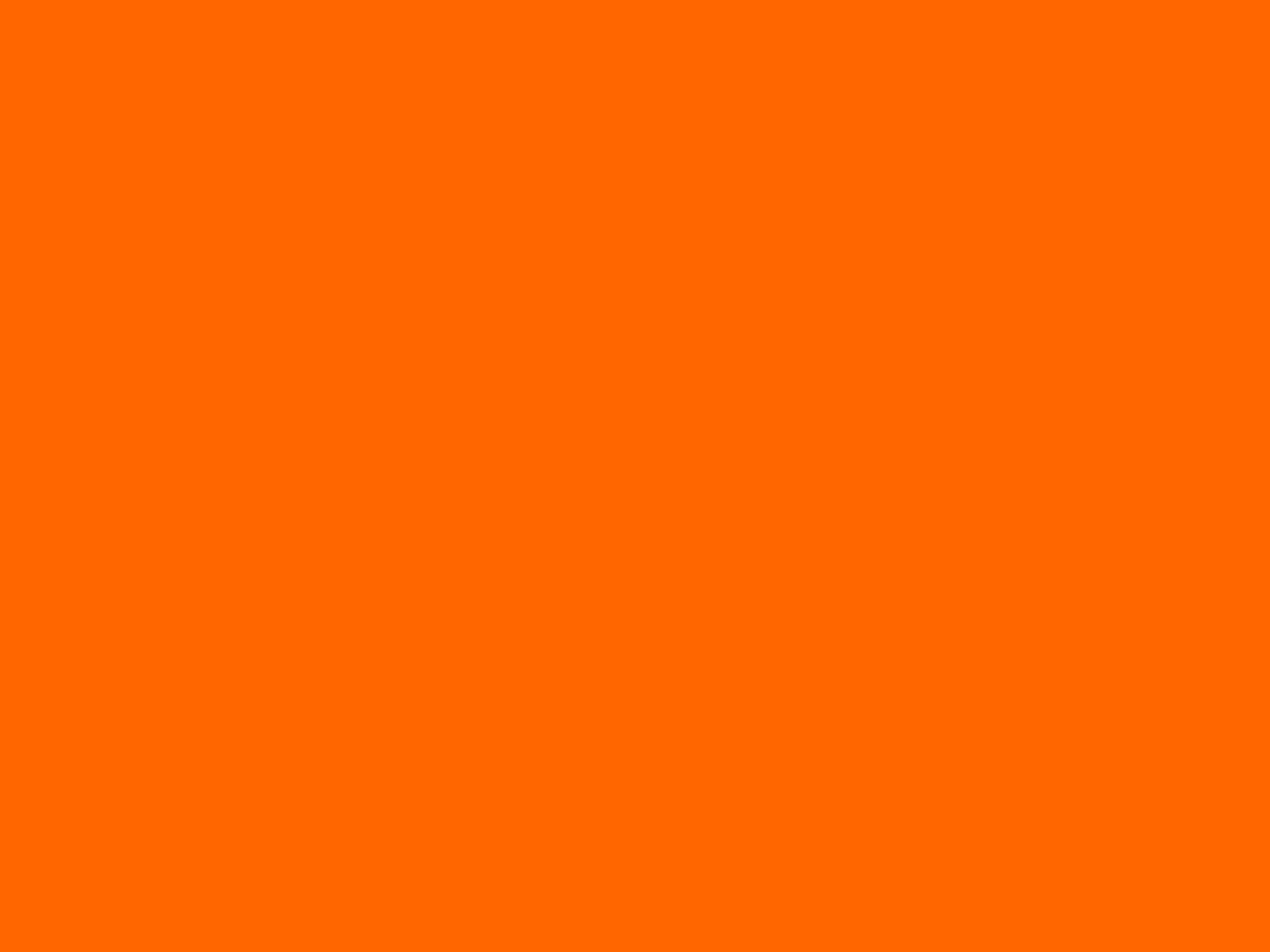

Supplement: IPE895028 MagicWandMovie3 - Supplemental material for Dynamic Amodal Completion Through the Magic Wand Illusion [file IPE895028_MagicWandMovie3.ppsx]
